# Exploring the biomechanical complexity of glioblastoma spheroids and organoids with co-localized Brillouin and Raman microspectroscopy

**DOI:** 10.1016/j.bbrep.2025.102227

**Published:** 2025-08-28

**Authors:** Roberta Galli, Jan Rix, Tina Leonidou, Katrin Kirsche, Edmund Koch, Achim Temme, Ilker Y. Eyüpoglu, Ortrud Uckermann

**Affiliations:** a Medical Physics and Biomedical Engineering, Faculty of Medicine, TU Dresden, Dresden, Germany; b Department of Neurosurgery, Faculty of Medicine and University Hospital Carl Gustav Carus, TU Dresden, Dresden, Germany; c Clinical Sensoring and Monitoring, Department of Anesthesiology and Intensive Care Medicine, Faculty of Medicine, TU Dresden, Dresden, Germany

**Keywords:** Brillouin microscopy, Raman spectroscopy, Brain tumors, In vitro models, Label-free, Multiphoton microscopy

## Abstract

Brillouin microscopy allows mechanical investigations of biological materials at the subcellular level and can be integrated with Raman spectroscopy for simultaneous chemical mapping, thus enabling a more comprehensive interpretation of biomechanics. The present study investigates different in vitro glioblastoma models using a combination of Brillouin and Raman microspectroscopy. Spheroids of the U87-MG cell line and two patient-derived cell lines as well as patient-derived organoids were used. Brillouin microscopy provided maps of viscoelastic parameters, while Raman spectroscopy identified key biochemical components such as proteins, lipids, glycogen and cholesterol. Cluster analysis of the Raman spectra allowed the categorization of biochemical groups and the correlation of their Brillouin shift and bandwidth across the different glioblastoma models. The results showed that spheroids from the same cell line exhibited relatively homogeneous biomechanical properties, while differences existed between different cell lines. In contrast, organoids from the same patient exhibited greater mechanical and biochemical heterogeneity. Brillouin shift and bandwidth showed significant variation among Raman clusters, highlighting the need to consider biochemical composition in biomechanical assessments. The cytoplasmic protein cluster was biochemically and biomechanically consistent across models, while lipid- and glycogen-related clusters varied. The approach used in this study facilitates the interpretation of Brillouin data in heterogeneous biological systems and allows comparisons between different models. The results emphasize the need for multimodal analysis for correct interpretation of biomechanical measurements in complex tissues and for comparison between heterogeneous samples.

## Highlights

• Brillouin/Raman microspectroscopy correlates tissue mechanics and chemistry.
• Biomechanical mapping of glioblastoma models reveals tissue heterogeneity.
• Comparison of viscoelastic parameters of tissue components is possible across models.
• Multimodal analysis facilitates the interpretation of biomechanics in neuro-oncology.

## Graphical abstract

**Figure:**
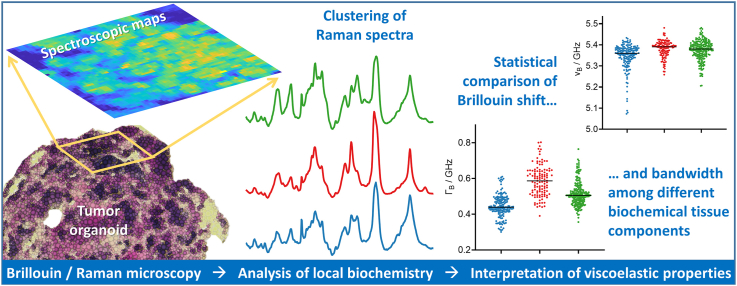

## 1 Introduction

The significance of biomechanics in tumor biology, and more specifically in neuro-oncology, is increasingly recognized [1,2]. Tumors exhibit biochemical and biomechanical properties that differ from those of normal tissue, while mechanical properties of tumor cells affect their metastatic potential [3]. Therefore, targeting tumor biophysics may support the future development of mechano-based therapeutic approaches [4]. Glioblastomas (GBM) are a malignant type of brain tumors with highly infiltrative growth and poor prognosis. The mechanical properties of GBM have been investigated across various length scales using different techniques ranging from magnetic resonance elastography to atomic force microscopy, which revealed high heterogeneity in GBM tumor stiffness and led to contradictory results [1]. Therefore, further studies are required to better understand the biophysical properties of tumor tissue components.

Brillouin microscopy is a non-contact optical technique that allows the determination of the viscoelastic properties of biological material at subcellular resolution [5]. It enables local biomechanical investigations without contacting the sample, while tight focusing of the excitation laser beam and confocal detection facilitate the analysis of cellular compartments [6].

Brillouin spectroscopy exploits the inelastic scattering of photons interacting with acoustic phonons in the sample and addresses the longitudinal modulus $M=M^{\prime }+iM^{\prime \prime }$ in the GHz frequency range. The storage (elastic) modulus $M^{\prime }$ and the loss (viscous) modulus $M^{\prime \prime }$ are related to the Brillouin shift $\nu_{B}$ and linewidth $\Gamma_{B}$ by the following equations:

$$M^{\prime }=\frac{\rho \lambda^{2}}{4n^{2}}{\nu_{B}}^{2}\;\text{and}\;M^{\prime \prime }=\frac{\rho \lambda^{2}}{4n^{2}}\nu_{B}\Gamma_{B}$$

where n is the local refractive index, ρ the mass density and λ the excitation wavelength [7]. In practice, Brillouin microscopy is used to reveal local changes in the biomechanics of cells and tissues without any prior knowledge of n and ρ [5,8], as the changes of density and square of refractive index compensate each other in many biological systems. Therefore, the Brillouin shift can be considered as an indicator of changes in stiffness of biological materials [9]. The Brillouin linewidth describes the viscous damping, but also depends on material heterogeneity (heterogeneous broadening). In soft and homogeneous matter with high hydration, Γ_*B*_ is proportional to the kinematic viscosity [10].

Brillouin spectroscopy can be integrated with Raman spectroscopy to perform simultaneous chemical-mechanical spectroscopy [11]. Since Raman spectroscopy is a biochemical analysis method that provides insights into a sample's composition (e.g., lipids, proteins, nucleic acids), it can serve as a reference to correlate biochemistry and biomechanics at the measurement position. The advantages of combined measurements have already been demonstrated in several studies on single cells [[12], [13], [14], [15]] and tissue [[16], [17], [18], [19], [20], [21], [22], [23], [24]]. Beyond general feasibility, it has been shown that the biochemical information in Raman spectra can facilitate the interpretation of the Brillouin data of heterogeneous biological media [25].

Previous investigations of Brillouin microscopy on GBM have been mainly devoted to cell cultures and especially on 3D cultures like spheroids, which simulate tumors more realistically than individual cells [26]. The intracellular mechanics of U87 glioblastoma cell lines have been measured in 2D and 3D cultures. It was found that the Brillouin shift correlates with pharmacological modulation of actin cytoskeleton, while the observed heterogeneity in cell shape closely correlates with the mechanical state of the cells [27]. Moreover, other studies showed that U87 cells in spheroids are stiffer than adherent cells [28].

The advantages of integrating Brillouin microscopy into the multimodal mapping of cells and tissues are undisputed [29]. Yet, investigations of correlative Raman-Brillouin microscopy in neuro-oncology are rare, and Brillouin microscopy studies on brain tumor tissue are lacking. The nervous tissue and brain tumors are inherently heterogeneous, comprising different cell types and variable extracellular matrix, which both affect the biomechanics of whole tissue [30]. Subcellular components like cytoskeleton, nucleus, cytoplasmic proteins and solutes determine the viscoelasticity of GBM cells, while extracellular matrix influences the mechanics of tumor tissue as a whole [31]. This complicates the interpretation of Brillouin measurements without local information about tissue components. Additionally, changes in refractive index and mass density do not always compensate each other, as in the case of lipid-rich structures [28], which makes the biochemical information even more important for correct interpretation of Brillouin data.

In this study, we explored the biomechanical heterogeneity of GBM models in vitro using the biochemical information of Raman spectroscopy as reference. Our objective was to improve the interpretation of Brillouin microscopy in heterogeneous systems and enable comparison across different systems and models. To this end, we compared among spheroids of the immortalized cell line U87-MG, spheroids of two primary cell lines and patient-derived GBM organoids, which recapitulate the histological and molecular heterogeneity of tumor tissue [32]. Raman spectroscopy was used to identify tissue biochemical components (e.g., proteins, lipids, etc.) and compare their Brillouin parameters across the different models.

## 2 Methods

### 2.1 Ethics statement

All patients provided written consent and the study was approved by the ethics committee of TU Dresden (EK 323122008).

### 2.2 Glioblastoma spheroids

U87-MG, HT18136 and HT18584 cells were grown in Neuropan basal medium + L-glutamine supplemented with 10 % Panexin basic, 1x Neuropan 27 supplement and 1x Neuropan 2 supplement (all PAN Biotech, Aidenbach, Germany). For preparation of spheroids, single cells were suspended in medium and cultured in T75 cell culture flasks in upright position for 5–12 d. Half of the medium was exchanged twice a week.

### 2.3 Glioblastoma organoids

The organoids were prepared following the protocol of Jacob et al. [32]. Fresh surgically resected human glioblastoma tissue was obtained in BrainBits Hibernate A medium, transferred to H + GPSA medium (Hibernate A, 1 × GlutaMax, 1 × PenStrep and 1 × Amphotericin B, all from Thermo Fisher Scientific, Darmstadt, Germany unless otherwise stated), washed, and cut into pieces sized 0.5–1 mm. Subsequently, it was washed with Hank's Balanced Salt Solution, and erythrocytes were removed using Red Blood Cell Lysis Solution (Miltenyi Biotec, Bergisch Gladbach, Germany). The tissue fragments were transferred to ultra-low attachment 6-well plates with 4 ml GBO medium (50 % DMEM:F12, 50 % Neurobasal, 1 × GlutaMax, 1 × NEAAs, 1 × PenStrep, 1 × N2 supplement, 1 × B27 w/o vitamin A supplement, 55 μm 2-mercaptoethanol (Merck KGaA, Darmstadt, Germany), 2.5 μg/ml human insulin (Merck KCaA)). Cultures were maintained at 37 °C with 5 % CO_2_, on a shaking platform set at 70 rpm, with a 75 % medium change every 48 h. After two weeks, organoids were cut to maintain their dimension below ∼1 mm and transferred to 96-well plates for further cultivation.

### 2.4 Brillouin and Raman micro-spectroscopy

The combined Brillouin-Raman system was already described in details elsewhere [28]. Shortly, the system uses a tunable diode laser stabilized at λ = 780.24 nm (TApro laser source with Doppler-free saturation spectroscopy stabilization module CoSy; TOPTICA Photonics, Gräfelfing, Germany). Two Bragg gratings (NoiseBlock; ONDAX Inc., Monrovia, CA, USA) and a doubly passed tunable Fabry–Pérot–Etalon with free spectral range of 15 GHz (LightMachinery, Nepean, Canada) were used to suppress the amplified spontaneous emission of the laser. The excitation light was coupled in a single-mode fiber and propagated to an upright reflection microscope (WITec alpha 300R; WITec, Ulm, Germany) customized with a dichroic beam splitter with edge at 785 nm to separate Rayleigh and Brillouin scattering from the Raman scattering. An N-Achroplan 40 × /0.75NA water-dipping objective was used to focalize the laser beam on the sample and collect the scattered light. The laser power on the sample was comprised between 20 and 25 mW. The Raman scattering was analyzed with a commercial spectrometer (UHTS 400; WITec GmbH, Ulm, Germany).

The Brillouin and Rayleigh scattering were propagated to the Brillouin spectrometer. Two Rubidium vapor cells (TG-ABRB-Q; Precision Glassblowing Inc., Englewood, CO, USA) were used to remove the Rayleigh scattering. A two-stage VIPA set-up was used for the spectral analysis of Brillouin scattering. The orthogonally arranged VIPAs had a free spectral range of FSR1 = 15 GHz and FSR2 = 21.6 GHz (LightMachinery Inc., Nepean, Canada). A CCD camera (iDUS 420A-BR-DD; Andor Technology, Belfast, UK) with a magnification objective (InfiniProbe TS-160; Infinity Photo-Optical Company, Centennial, CO, USA) was used to acquire the Brillouin spectra. The average spectral resolution was 44 MHz/pixel, calculated considering that the distance between the Stokes and anti-Stokes Brillouin bands of water was 230 pixels on the CCD. In order to calibrate the Brillouin frequency, the Brillouin signal of methanol was generated in a reference beam path built in parallel to the microscope. The Stokes and anti-Stokes Brillouin band of methanol (ν_B_ = 3.81 GHz) were acquired in each spectrum superimposed to the bands of the sample.

Spheroids and organoids were measured in Petri dishes in their culturing medium. Bidimensional maps were acquired by raster-scanning the samples, with a single-point integration time of 0.2 s. Combined Brillouin/Raman line maps were acquired with an acquisition time per point of 45 s on spheroids and 150 s on organoids.

The system-related uncertainty was evaluated on a sucrose solution with a shift ν_B_ = 5.5 GHz and Γ_B_ = 0.43 GHz, using the same acquisition parameters as for tissue mapping. With an acquisition time of 0.2 s, the standard deviation was 21 MHz for ν_B_ and 51 MHz for Γ_B_ (average values obtained from five maps with 100 points each). With an acquisition time of 45 s, standard deviations of 5 MHz and 18 MHz were found for ν_B_ and Γ_B_, respectively (average values obtained from five maps with 100 points each). The enhanced repeatability by very long acquisition time is due to complete noise suppression in the Brillouin spectra.

### 2.5 Data analysis and statistics

A self-written code in Matlab (MathWorks Inc., Natick, MA) was used to fit the Brillouin bands as Lorentzian functions and retrieve the frequency shift, the linewidth (calculated as full width at half maximum - FWHM) and the intensity of the Brillouin band. The code is described elsewhere [28]. A spectrum of water with Lorentzian fitting is exemplarily shown in Supp. Fig. S1.

The linewidth was corrected for the system broadening, which was retrieved by measuring the FWHM of the Rayleigh band prior to experiments. Considering the measured Brillouin bands as the result of the convolution of Lorentzian curves and thus having FWHM equal to the sum of its Lorentzian components, the corrected FWHM of the Brillouin band of the sample was achieved by subtracting the FWHM of the Rayleigh band from the measured FWHM of the measured bands. The FWHM of the Rayleigh band was 400 MHz for spheroids experiments and 330 MHz for organoids experiments. Improvements of the set-up and of alignment of the Brillouin spectrometer was performed between the two measurement campaigns and justifies the different bandwidth.

Raman spectra were processed in Matlab performing a baseline correction to remove the background (function: msbackadj). Area normalization (function: msnorm) was applied prior to cluster analysis (k-means cluster analysis with Euclidean metric, function: kmeans). The Matlab function 'evalclusters' was used to determine the optimal number of clusters for each sample type, and the results were compared using different criteria (Silhouette, Davis–Bouldin and Calinski–Harabasz). This was then verified using a Silhouette analysis (Matlab function: silhouette), and the results were compared with the biochemical information provided by the centroid Raman spectra to determine their meaningfulness. This procedure is illustrated for U87-MG spheroids in Supp. Fig. S2.

Statistics were calculated using Prism 10.2.3 (GraphPad Software, Inc., San Diego, CA, USA). Statistical methods are stated in the Results section. Differences were considered significant if P < 0.05.

### 2.6 Multiphoton microscopy

Label-free multiphoton microscopy was performed immediately after the spectroscopic measurements, using a multimodal laser scanning system producing coherent anti-Stokes Raman scattering (CARS) tuned on the Raman band at 2785 cm^−1^ to visualize lipids, two-photon excited fluorescence (TPEF) and second harmonic generation (SHG). The system is described elsewhere [33]. For imaging, spheroids and organoids were placed on a glass slide in a drop of culturing medium and a coverslip was applied on top. A W Plan 20 × /1.0 NA Apochromat Zeiss objective was used. On spheroids, CARS and SHG were acquired in transmission and TPEF in reflection, while all three signals were acquired in reflection on organoids. RGB images were obtained combining CARS in the red channel, TPEF in the green channel and SHG in the blue channel.

### 2.7 Reference staining

After imaging, spheroids and organoids were fixed in 4 % formaldehyde solution at 7 °C for 1–5 days and then were freeze-protected in ascending sucrose solution (10 % and 30 % for 24 h each). The samples were embedded in cryomedium, frozen on dry ice and then stored at −80 °C. Frozen tissue sections were prepared at a thickness of 10 μm and stored at −20 °C.

Hematoxylin and eosin (HE) staining: The sections were washed in distilled water and incubated with Mayer's hematoxylin/hemalum for 3 min. After washing in distilled water, the tissue was briefly destained in HCl-ethanol. Washing with tap water for 5 min was followed by 3 min of staining with eosin (1 % eosin G in 80 % ethanol). The sections were dehydrated in increasing ethanol concentrations, cleared in xylene and mounted with Entellan.

Periodic Acid-Schiff (PAS) Staining: The sections were rinsed in distilled water and incubated in 0.5 % periodic acid for 3 min. After washing for 3 min, they were incubated in Schiff's reagent for 20 min and then rinsed for 5 min. Counterstaining was performed with Mayer's acid hematoxylin for 5 min (all products from the staining kit Product-N. 12153, Morphisto GmbH, Offenbach, Germany). The sections were then rinsed in tap water for 3 min, dehydrated in increasing ethanol concentrations, cleared with xylene, and mounted with Entellan.

Ki67 staining: The sections were fixed in methanol-acetone solution at −20 °C, and then dried for 30 min. For heat-induced epitope recovery, cryosections were incubated in citrate buffer (pH = 6.0) for 40 min in a steamer. Peroxidase blocking was performed with 0.3 % hydrogen peroxide-methanol solution for 10 min. The block serum was added (0.3 % Triton X-100 in 1 % bovine serum albumin solution), followed by a 1 h incubation. The primary antibody (Novocastra monoclonal mouse antibody Ki67 antigen, Leica Biosystems) was added to the blocking serum (1:200), and the sections were incubated overnight. After washing in PBS, the sections were incubated with the secondary antibody (Histofine Simple Stain MAX PO (M), Nichierei Biosciences), followed by colorimetric detection of the antibody signal (HistoGreen Kit, Linaris). The samples were washed again in PBS and counterstained with nuclear red. After dehydration in ethanol and xylene, the sections were mounted with Entellan.

## 3 Results

### 3.1 Glioblastoma spheroids

For each cell line (U87-MG and patient-derived cell lines: HT18136 and HT18584), we analyzed n = 18 spheroids, acquiring Brillouin maps composed of 50 × 50 points with 2 μm step (n = 8) and Brillouin-Raman lines of 1 × 50 points with a 1 μm step (n = 10).

**Fig. 1:**
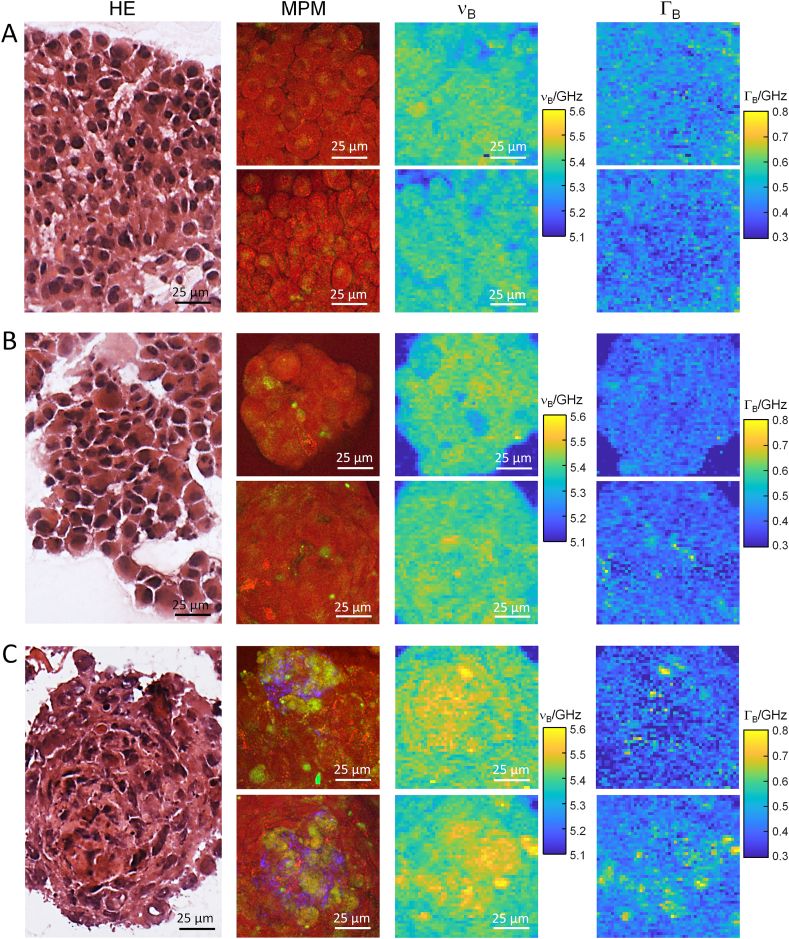
Glioblastoma spheroids. A) U87-MG. B) Primary cell line HT18136. C) Primary cell line HT18584. HE staining, multiphoton microscopy (MPM) images and maps of Brillouin shift (ν_B_) and bandwidth (Γ_B_) are shown from left to right for each cell line. Color coding of MPM channels: CARS: red, TPEF: green, SHG: blue.

We found a high degree of inter- and intra-spheroidal histological homogeneity for the U87-MG spheroids, in agreement with the high phenotypic stability of this cell line. Fig. 1 A exemplarily shows the results of measurements on two U87-MG spheroids, which are representative for all other spheroids. The HE staining shows a compact structure composed of cells with large nuclei. Nuclei with prominent nucleoli are visible in the multiphoton images, and CARS reveals small intracellular lipid droplets in almost all cells. In the maps of Brillouin shift ν_B_, cells can be discerned especially at the border of the spheroid. In contrast, the maps of the Brillouin bandwidth Γ_B_ do not reveal any structure.

The HT18136 spheroids are similar to U87-MG regarding their morphology as shown by histology and multiphoton microscopy, and their viscoelastic properties as shown by Brillouin spectroscopy (Fig. 1 B). A strong accumulation of lipid droplets in some cells is visible in the multiphoton images and may account for the spots with higher ν_B_ and slightly increased Γ_B_. In contrast, HT18584 spheroids (Fig. 1 C) show a different structure. Besides the presence of lipid droplets, they show a collagenous core, corresponding to the SHG-active regions that are visible in the multiphoton images. The Brillouin shift and bandwidth are locally increased, and these regions may eventually correspond to the collagenous one.

**Fig. 2:**
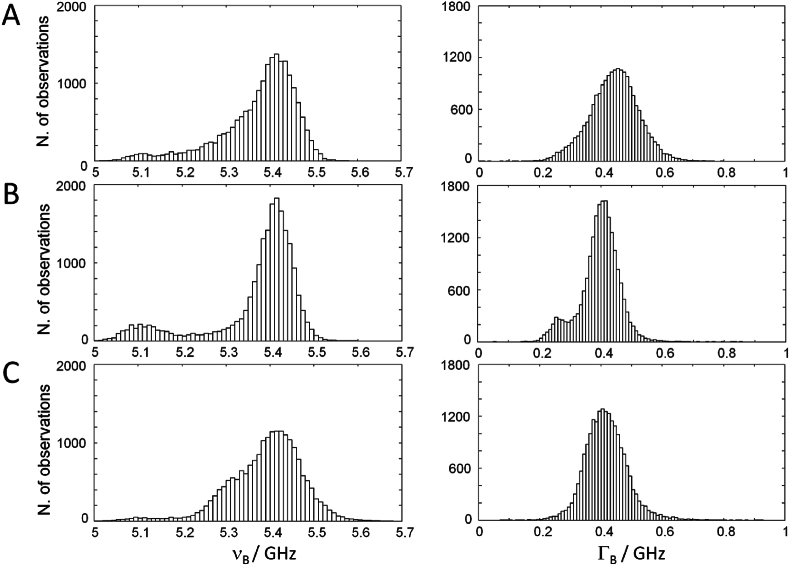
Distribution of Brillouin shift and FWHM of glioblastoma spheroids. A) U87-MG. B) Primary cell line HT18136. C) Primary cell line HT18584. Each frequency histogram contains 20,000 observations (n = 8 spheroids for each cell line, one map for each spheroid, with 2500 points per map). Bin: 50 MHz.

The histograms of the distribution of ν_B_ and Γ_B_ are shown in Fig. 2 for all analyzed spheroids (for each cell line: eight maps of 2500 points each, shown in Supp. Fig. S3). The distributions of ν_B_ display a maximum at 5.41 GHz for all cell lines, while the values near 5.1 GHz represent the medium. The distributions of Γ_B_ are symmetric, that of U87-MG spheroids being centered at 0.44 GHz, and that of spheroids from both primary cell lines at 0.40 GHz. The values of Γ_B_ close to 0.25 GHz represent the medium. The scatter plots of bandwidth vs. shift show poor correlation, indicating the presence of heterogeneous broadening (Supp. Fig. S4); some degree of correlation exists only for the medium and highly hydrated part of the tissue with approximately ν_B_ < 5.3 GHz. This is consistent with previous findings on biological tissue [34]. The system-related uncertainty on ν_B_ and Γ_B_ (see section 2.4) only accounts for a minor contribution to the observed spreading.

**Fig. 3:**
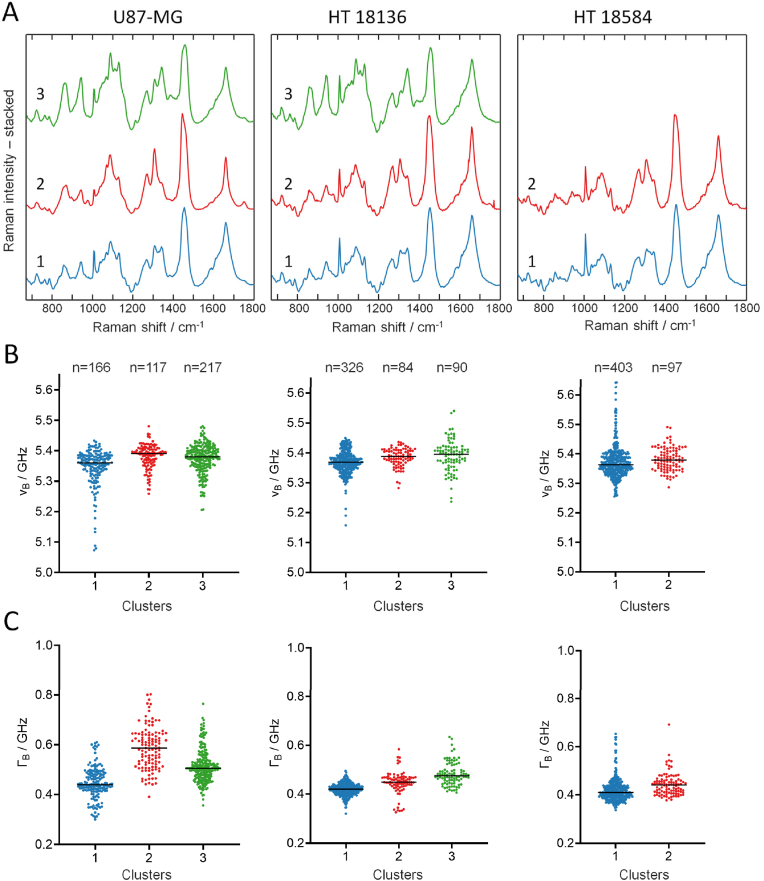
Cluster analysis of Raman spectral datasets of glioblastoma spheroids. A: Centroid Raman spectra. B: Brillouin shift for the clusters found by analysis of the Raman spectra. C: Brillouin bandwidth for the same clusters. The median values are indicated.

To gain insight into the biochemical properties that underlie the different Brillouin data, we used the combined Brillouin and Raman information obtained in the line maps (examples are shown in Supp. Fig. S5). We used cluster analysis of the Raman spectra of each cell line to retrieve biochemical groups and compare their Brillouin parameters. All measured points were used in the analysis, that is, 500 point-measurements for each cell line. The centroid spectra of clusters are shown in Fig. 3 A. For U87-MG and HT18136 spheroids, three clusters describe the major components, while only two clusters are sufficient to describe the cell line HT18584. Clusters 1 of all cell lines were attributed to cell proteins in agreement with previous experiments on GBM cells [28]. Clusters 2 are all characterized by high Raman intensity at 1440 cm^−1^, which is typical of lipids [35]. They account for the lipid droplets visualized by CARS in the multiphoton microscopy images of Fig. 1. Cluster 3 is characterized by two Raman bands at 865 and 942 cm^−1^, which are attributed to glycogen, while all other bands are assigned to proteins [36]. The presence of glycogen was confirmed by PAS staining (Supp Fig. S6 A). Therefore, cluster 3 was interpreted as a mixture of proteins and glycogen. This assumption was confirmed by subtracting the centroid spectra of cluster 1 from the one of cluster 3 and comparing the obtained difference spectrum with a reference spectrum of pure glycogen (Supp Fig. S6 B). When comparing the centroid spectra of the corresponding clusters between different cell lines, it is evident that the spectral profiles of clusters 1 (proteins) and 3 (proteins and glycogen) are almost identical. In contrast, the spectral profile of cluster 2 (lipids) shows small differences at around 1100 and 1300 cm^−1^.

The Brillouin shift and bandwidth of the groups found by cluster analysis of the Raman spectra were retrieved (Fig. 3B and C) and statistically analyzed. The shift and the bandwidth of cluster 2 (lipids) and of cluster 3 (proteins and glycogen) are larger compared to cluster 1 (proteins). Brillouin shift differences among clusters are statistically significant for the U87-MG and HT18136 spheroids (Welch ANOVA test, P < 0.001), but not for the HT18584 spheroids (Welch *t*-test, P = 0.0531). Difference of bandwidth are partly more evident and statistically significant for all three cell lines (same tests used for the shift, i.e., Welch ANOVA test and Welch *t*-test, P < 0.001 in all cases). Results of the multiple comparison tests between groups are reported in Supp. Table S1 for both Brillouin shift and bandwidth, with P values and group dimensions. There, it is possible to see that lower P are found for Γ_B_ than for ν_B_, in agreement with the fact that the bandwidth is known to be more sensitive to changes [37,38].

Overall, the median shifts and bandwidths of clusters are quite consistent across all cell lines, with only Γ_B_ of cluster 2 (lipids) of the U87-MG spheroids larger compared to the other spheroids. This explains the different distribution of ν_B_ of U87-MG already observed in the histograms of Fig. 2. The Brillouin Loss Tangent BLT = Γ_B_/ν_B_ = M’’/M’ of the biochemical clusters provided analogous differences (Supp. Fig. S7). The changes in the BLT are driven by changes in the bandwidth, as found in other studies on biological tissues [34]. Since the BLT is independent on the refractive index and density, it confirms that viscoelastic properties of biochemical clusters are different.

To better identify the sources of Brillouin band broadening, the correlation between ν_B_ and of Γ_B_ was examined for each cluster (Supp. Fig. S8). A certain degree of correlation between Brillouin shift and bandwidth is found for certain clusters only when both values are high, suggesting that few viscosity changes can be retrieved above the dominating heterogeneous broadening. In this case too, it is worth noting that the system-related uncertainty is much smaller than the observed spreading of data (see section 2.4). The presence of heterogenous broadening is explained by actual spatial resolution, which is defined by the phonon mean free path, which is typically larger than the optical resolution attained with high NA objectives. [39]. For example, the phonon mean free path assumes values of ∼ 2.5 μm at 532 nm excitation in soft materials or in water [10,39]. Therefore, the dimensions of most intracellular structures are in the same range of the phonon mean free path (e.g. cell organelles) or smaller (e.g. actin filaments). In both cases, the shape of Brillouin band is increased as consequence of the presence of partly overlapping peaks or by phonon confinement effects [10,40]. The two specific cell features found in spheroids – i.e., lipid droplets and glycogen granules - have a size that is below or in the same order of the actual resolution. Lipid droplets in GBM are typically 1–2 μm [41], in agreement with information from CARS images, and therefore in the same size range as the spatial resolution. Glycogen granules have a size <0.2 μm [42], thus much smaller than the resolution. This explains also the mixing of glycogen and protein Raman bands obtained for cluster 3.

### 3.2 Glioblastoma organoids

**Fig. 4:**
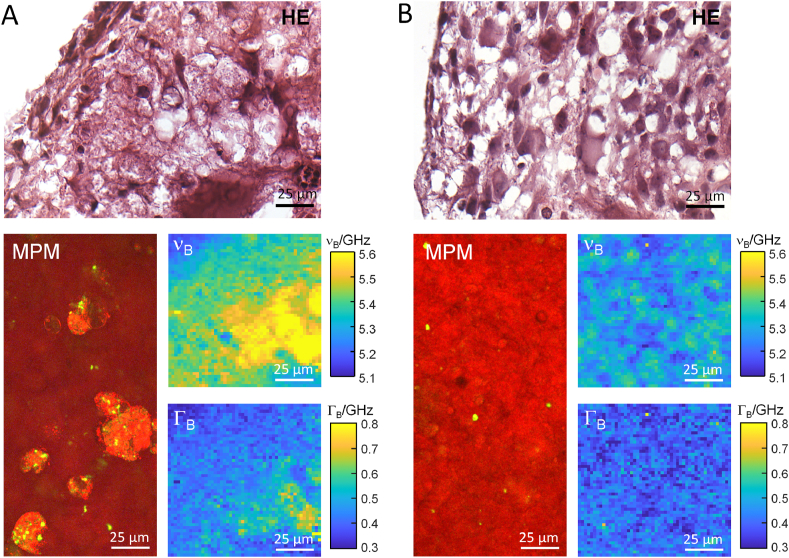
Glioblastoma organoids. A: Organoid measured at week 4. B: Organoid measured at week 8. HE staining, multiphoton microscopy (MPM) images and maps of Brillouin shift (ν_B_) and bandwidth (Γ_B_). Color coding of MPM channels: CARS: red, TPEF: green, SHG: blue.

Histology (Fig. 4) and Ki67 immunohistochemistry of sections (Supp Fig. S9 A) showed some intra-organoid heterogeneity and the presence of a necrotic core in large organoids due to diffusion limits. Ki67 staining confirms that the peripheral regions are highly proliferative and thus viable. The spectroscopic measurement depth did not exceed 50–60 μm, and therefore all measurements were located within the vital, proliferating tissue. The necrotic core lies beyond the measurable depth and was always excluded from analysis, which was restricted to the properties of viable tumor tissue and aimed to sampling of comparable, biologically relevant regions across organoids. MPM imaging of fresh organoids confirmed large inter-organoid heterogeneity, as exemplarily shown in Fig. 4 (more examples are shown in Supp Fig. S9 B). As MPM was performed on fresh organoids and its penetration depth is similar to the Brillouin measurements, MPM images captured comparable superficial regions.

**Fig. 5:**
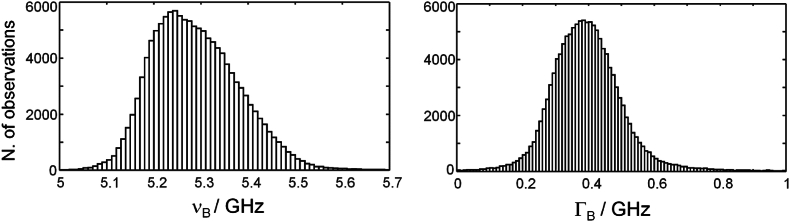
Distribution of Brillouin shift and FWHM of glioblastoma organoids. The frequency histograms contain 130,000 observations (52 maps with 2500 points each, acquired on n = 16 organoids. Bin: 0.05 GHz.

We analyzed n = 16 organoids by Brillouin microscopy, performing maps with 50 × 50 points and 2 μm step to capture the local variability (three or four maps per organoid) and combined Brillouin/Raman lines (20 points, 5 μm step; one line map per organoid). High heterogeneity was observed in the Brillouin maps (Fig. 4 and Supp. Fig. S10). Rather large structures with increased Brillouin shift and width qualitatively matched the foam cells visible in the MPM images. The histograms of ν_B_ and Γ_B_ (Fig. 5) are broader compared to spheroids, reflecting the higher heterogeneity of organoids. The maximum of ν_B_ histogram is observed at 5.25 GHz (lower frequency compared to spheroids), while the Γ_B_ histogram is centered at 0.40 GHz and, therefore, the same value as for the spheroids of patient-derived cell lines. The scatter plot of ν_B_ vs. Γ_B_ shows a rather poor correlation, indicating presence of heterogeneous broadening (Supp. Fig. S11).

**Fig. 6:**
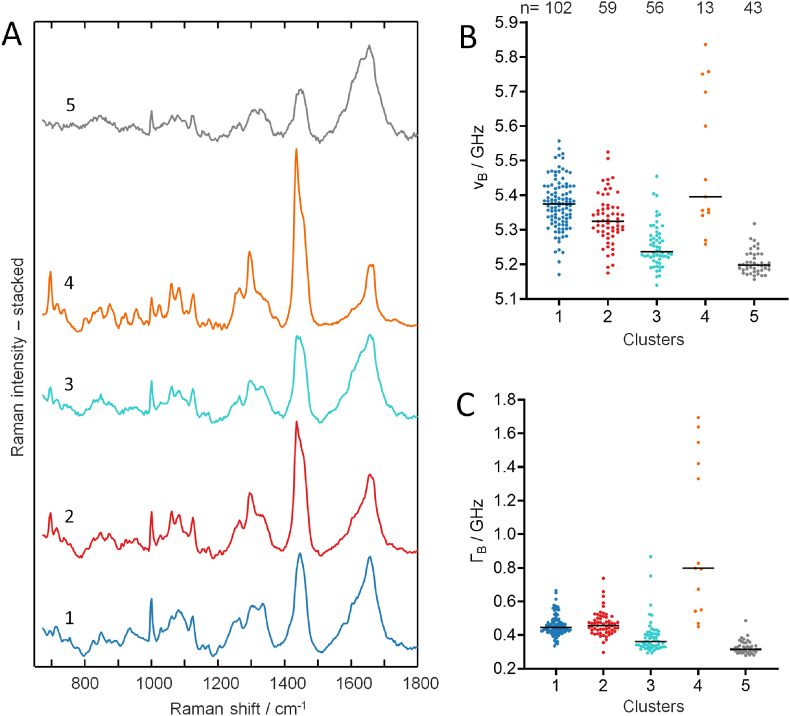
Cluster analysis of organoids. A. centroid Raman spectra of the five clusters. B: Brillouin shift for the five clusters found by analysis of the Raman spectra. C: Brillouin bandwidth for the five clusters found by analysis of the Raman spectra. The median values are indicated. Biochemical assignment of clusters: 1: proteins, 2: lipids, 3: proteins, 4: cholesterol-rich lipids, 5: highly diluted proteins (intercellular spaces).

The organoids’ biochemistry was retrieved from the combined Brillouin/Raman line maps (see examples in Supp. Fig. S12) and the Raman spectra of all organoids were subjected to cluster analysis (Fig. 6). All point measurements acquired in the line maps were used in the analysis. Compared to cell lines, more clusters were required to describe all biochemical components; for instance, two protein clusters (clusters 1 and 3) and two lipid clusters (clusters 2 and 4) were found, plus an additional cluster (cluster 5) with weak bands of proteins, which might be interpreted as intercellular spaces. A sixth cluster containing 47 points with Raman spectrum of pure medium or bad Raman spectrum without bands was also found. It was not further considered in the analysis, which was then conducted on five clusters with a total of 273 points.

As shown in Fig. 6A, the main difference between the protein clusters involve the Amide III region in the spectral range 1250–1350 cm^−1^ and in the C–N and C–C stretch region at around 1100 cm^−1^. Cluster 4 represents cholesterol-rich lipids, as can be inferred by the intensity of the characteristic cholesterol ring vibration at 700 cm^−1^ [35,36].

As shown in Fig. 6B and C, the biochemical clusters display different viscoelastic properties. The differences among groups are significant for both Brillouin shift and bandwidth (Welch Anova test, P < 0.001). The results of multiple comparison tests with P values reported in Supp. Table S2. Similar differences were found also for the BLT (Supp. Fig. S13). Some correlation shift-width (Supp. Fig. S14) could be found for all clusters except cluster 5 (intercellular spaces).

### 3.3 Comparison of glioblastoma cell lines and organoids

The median values of Brillouin shift and bandwidth are resumed in Table 1. The cluster 1 of proteins display very similar parameters in all models. The Raman centroid spectra are very similar too, being almost identical for HT18584 spheroids and organoids. Cluster 1 of all models was interpreted as cytoplasm by comparison of the centroid Raman spectrum with previous experiments on single GBM cells in culture [28]. Therefore, small (but partially statistically significant) differences in the shift of cluster 1 across models as shown in Supp. Fig. S15 might reflect cytoskeletal changes. The variations of cluster 1 bandwidth across models are slightly larger. The viscosity of U87-MG and organoid cell cytoplasm is likely higher compared to patient-derived cell lines, assuming that the heterogeneous broadening is similar for all cell lines.

**Table 1:** Median values of Brillouin shift and bandwidth for the biochemical groups found with the cluster analysis of Raman spectra of GBM spheroids and organoids.

	νB	ΓB	νB	ΓB	νB	ΓB	νB	ΓB	νB	ΓB
	Cluster 1 cytoplasm		Cluster 2 lipid droplets		Cluster 3 proteins + glycogen					
U87-MG	5.36	0.44	5.39	0.59	5.38	0.51				
HT18136	5.37	0.42	5.39	0.45	5.39	0.47				
HT18584	5.36	0.41	5.38	0.44	–	–				
	Cluster 1 cytoplasm		Cluster 2 lipid droplets		Cluster 3 proteins		Cluster 4 cholesterol-rich lipids		Cluster 5 intercellular spaces	
Organoids	5.37	0.45	5.32	0.46	5.24	0.36	5.40	0.80	5.20	0.32

The cluster 2 of lipids was found in all models and was attributed to lipid droplets, which were confirmed by MPM in spheroids as well as in organoids. This cluster is not homogenous from both the biochemical and the biomechanical point of view across the different models. In spheroids, the higher Brillouin shift and bandwidth of this cluster compared to cytoplasmic proteins of cluster 1 does not likely reflect biomechanical changes. The Lorentz-Lorenz relation, which predicts that the refractive index squared (n^2^) scales with the mass density (ρ), so that variations in these parameters cancel each other out to a good approximation [38], does not apply to lipids [28]. Other groups have also investigated this phenomenon in detail and shown that the true longitudinal modulus of lipid droplets is actually lower than that of the cytoplasm when both refractive index and absolute density are considered [8]. Therefore, higher Brillouin shift of lipids cannot be interpreted as an increased stiffness when comparing to proteins. In organoids, however, the Brillouin shift of lipid cluster 2 is lower than that of protein cluster 1, indicating lower stiffness. Furthermore, different viscoelastic properties are indicated by the BLT.

The cluster 3 of spheroids representing a mixture of glycogen and proteins is characteristic of U87-MG and HT18136. Massive accumulation of glycogen in U87-MG spheroids was already observed [43] and is upregulated especially in hypoxic conditions [44]. Higher Brillouin shift of this cluster might be explained with the fact that glycogen is stored in solid granules within the cell cytoplasm [42]. Reference measurements of biomechanics of human glycogen granules are not available at our best knowledge. Some AFM measurements were done only on phytoglycogen particles, providing a Young modulus in the order of 200–600 kPa [45], which is one to two orders of magnitude larger compared to nervous cells and brain tumors [[46], [47], [48]]. Because of the very small dimension of glycogen granules, the increase of Brillouin bandwidth compared to cell cytoplasm is likely to be attributed to the heterogeneous broadening, as already explained in section 3.1.

Cluster 4, attributed to cholesterol-rich lipids, was found only in organoids. Its high Brillouin shift and bandwidth in comparison to protein clusters are also affected by the increased refractive index and decreased mass density. In comparison to lipid clusters, different viscoelastic properties are likely present. Considering a density of ρ = 0.8932 g/ml and refractive index n = 1.409 for lipid droplets [8], and general reference values of ρ = 1.06 g/ml and n = 1.525 for cholesterol, then the difference in the contribution of the term ρ/n^2^ to the Brillouin shift is as low as 1 %, and does not justify the very high measured shifts. These might rather reflect that cholesterol forms solid crystals, which were in fact sporadically visualized by MPM microscopy in organoids (Supp. Fig. S9).

## 4 Discussion

The biomechanics of brain tumors is a rapidly growing field of research. Several studies have shown that biomechanics is related to tumor malignancy, invasion properties and molecular profile. AFM allowed differentiation between WHO grade II, III, and IV astrocytomas, thus distinguishing between different degrees of malignancy [49]. A correlation between the strength of the extracellular matrix and the aggressiveness of brain tumors based on changes in mechanosignaling has been identified [50]. Durotactic stimuli are recognized as a critical factor for glioma cell migration [51]. It is known that the structure, motility, and proliferation of glioma cells are influenced by the biomechanical properties of the tissue [52,53]. However, experiments with patient-derived primary glioblastoma cell lines have shown significant variability between patients in their responses to biomechanical stimuli [54]. Different prognostic markers were found to be associated with differences in stiffness as measured by AFM, with IDH1 mutant cells being stiffer than IDH1 wild-type, and CD44-negative IDH1wt cells stiffer than CD44-positive IDH1wt cells [55].

Actin is known to regulate the mechanotransduction in GBM and is involved in cell migration. Cytoskeletal remodeling is emerging as a hallmark of the adaptive response of GBM cells to ECM compositional alterations and stiffening and may sustain invasion [56]. Recently, it has been shown that disruption of actin cytoskeleton can be detected by a decrease in the Brillouin shift, which correlates with actin fluorescence measurements [57]. Therefore, Brillouin microscopy is a suitable tool to selectively study the biomechanics of tumor cells and ECM, and to address changes in cell stiffness upon cytoskeleton remodeling.

However, it is important to note that Brillouin spectroscopy probes viscoelastic properties at high frequency and it is therefore not always equivalent to the analysis of the elastic modulus at low frequency as performed by AFM, micro-pipetting or micro-indentation. It is not possible to establish a theoretical correlation between these moduli for biological matter. Nevertheless, it was shown that the changes in the Brillouin shift correlate with changes in the elastic modulus [5,58], and empirical relationships between changes in the Brillouin shift and changes in the stiffness obtained using traditional rheological methods were identified [27]. Moreover, the aforementioned dependence of the Brillouin shift from density and refractive index further complicates the extraction of biomechanical information from Brillouin measurements.

We have previously shown that GBM spheroids have higher Brillouin shifts in comparison to adherent cells [28]. Here, we did not find a further increase in shift from spheroids to organoids, but rather increasing variation of Brillouin parameters. Cluster analysis of Raman spectra identifies biomechanical groups and allows comparison between different models. Despite biological differences between immortalized and patient-derived models, the comparable tissue architecture and consistent measurement conditions allow for a robust and meaningful comparison of Raman-derived biochemical clusters. Both GBM spheroids and organoids are three-dimensional tissue-like constructs that share comparable microscale architecture consisting of densely packed cells, have cell–cell interactions and ECM-like environments. As a result, the Raman measurement conditions (e.g., laser penetration depth and signal attenuation) are influenced by similar physical constraints in both systems. This ensures that groups built by clustering of Raman spectra reflect underlying biochemical signatures.

We found that spheroids of the same cell line are biochemically and biomechanically homogeneous. This was expected, as spheroids are clonal spheres of genetic identical cells; thus, they are highly reproducible and are available for high throughput analysis. Our results are in agreement with the findings of other research groups, which showed that U87 cells in spheroids exhibit less mechanical heterogeneity than cells in monodisperse 3D cultures, suggesting mechanical cooperation among the cells forming a single spheroid [27]. However, we found differences among primary cell lines. This is not surprising, as it has been already shown that patient-derived GBM glioblastoma cells exhibit distinct biomechanical profiles [31].

Organoids like used in this study are derived from patient material and consist of multiple cell types in a 3D organotypic structure, and are considered to mimic the biological features of the patient's tumor including response to therapy [59]. However, heterogeneity might change during cultivation [60]. Organoids are considered to bridge the gap between animal models and clinical studies. The increasing number of clusters required to describe organoids compared to spheroids highlights the higher complexity of tumor tissue. The Brillouin loss tangent, which does not depend on the refractive index n or mass density ρ [38], suggests that mechanical properties are the main contributors to the observed changes.

We obtained consistent similarities and differences among GBM models not only in the Brillouin shift, but also in the bandwidth, suggesting differences in viscosity. The influence of viscosity on mechanotransduction and disease has been less validated in mechanobiology compared to stiffness [29], although some studies have shown that cells are sensitive to viscosity cues as well [61]. Brillouin line shape analysis is highly relevant for the comparison of cells under physiological and pathological conditions: following oncogene expression, cells showed a strong decrease in apparent viscosity, which was explained by a general modification of the cytoskeletal properties and could favor their spreading in tissues [13].

Both spheroids and organoids are 3D in vitro tissue models that can be monitored over days and weeks. While in vitro spheroid and organoid models provide valuable platforms for investigating GBM biomechanics, it is important to contextualize their ability to recapitulate the mechanical characteristics of in vivo GBM tissue. These 3D models reproduce several key features of the tumor microenvironment, including cell–cell and cell–matrix interactions, spatial heterogeneity, and the development of gradients in nutrients, oxygen, and metabolic by-products. However, both are simplifications of the native tumor environment. Most notably, they lack contributions from the surrounding brain parenchyma, vasculature, immune components, and the full range complexity and stiffness gradients of ECM present in vivo. Alternative options might be embedding of tumor cells or samples in (specifically engineered or functionalized) hydrogels [62] or tumor-on chip models [63]. Moreover, investigation of fresh human brain tumor samples might enable to approximate the in vivo setting, hence with the disadvantage of having only a limited time window for investigation of tumor biomechanics on one experimental day.

The presented analytical approach based on analysis of biomechanical properties of biochemical components retrieved by Raman spectroscopy may be extended to the study of heterogeneous tissue samples from patients. However, an improvement in the Raman spectral quality and a further refinement of the analysis of the Raman spectra are likely required for distinguishing the cellular from the extracellular proteins and collagens, in order to address the specific biomechanics of the tumor extracellular matrix. This is of primary importance towards analysis of brain tumor biopsies, as some studies already underpinned the role of ECM in brain tumors. AFM studies have shown that brain tumors are stiffer than normal brain tissue, consistent with the profound alteration of brain ECM within malignant primary brain tumors [64]. While the stiffness of tumor ECM may increase, tumor cells – and especially invading cells – are generally soft, as seen in other types of cancers [65,66]. Therefore, it is essential to consider the biomechanics of the tumor ECM, tumor cells within the solid tumor, and invading cells separately in order to gain a comprehensive understanding of the complex mechanism underlying brain invasion.

Because Brillouin signals are influenced by biomechanical and biochemical factors, careful reference measurements and complementary analyses will be necessary for reliably interpreting the origin of observed changes in future applications of this technique, whether in vivo or ex vivo. In tissue, Brillouin frequency shifts may result from changes in stiffness, hydration, molecular composition, or structural organization. Disentangling these contributions is difficult. Therefore, reference datasets, such as those from healthy versus pathological tissue or from parallel biochemical staining and spectroscopic analyses, will be essential to establishing interpretive models. Furthermore, integrating this technique with others that provide low-frequency mechanical moduli will enable a more comprehensive investigation of GBM biomechanics. AFM can provide high-resolution topographical and mechanical maps of the cell surface and assess the mechanical properties of the entire cell depending on the indentation parameters [67]. Tip-enhanced Raman spectroscopy could be used to obtain biochemical information of the same spatial scale as AFM [68]. On the other hand, optical coherence elastography [69,70] and indentation [71] can be used to assess low-frequency viscoelastic properties of tumor tissue at larger scale. However, a comparative interpretation must carefully consider the different temporal and spatial scales, direction of applied forces, probed mechanical moduli, and other specific factors.

Looking ahead, technological improvements could significantly enhance the utility and interpretability of Brillouin microscopy. For instance, faster acquisition speeds could be achieved with different system designs [72,73], while machine learning [74] could aid in distinguishing subtle biochemical versus mechanical contributions across heterogeneous tissues.

In conclusion, the results demonstrated that spheroids of the same cell line exhibited relatively homogeneous biomechanical properties, while marked differences existed between different cell lines. Conversely, organoids from the same patient exhibited greater mechanical and biochemical heterogeneity. The application of cluster analysis to Raman spectra facilitated the categorization of biochemical groups and the correlation of their Brillouin parameters across the various GBM models. This strategy was found to be a useful for interpreting Brillouin data in heterogeneous biological systems and for facilitating comparisons across different models. The findings emphasize the necessity of multimodal analysis in neuro-oncology research, particularly for the accurate interpretation of biomechanical measurements in complex tissues and the comparison of heterogeneous samples.

## CRediT authorship contribution statement

**Roberta Galli:** Writing – review & editing, Writing – original draft, Visualization, Validation, Supervision, Methodology, Investigation, Formal analysis, Data curation, Conceptualization. **Jan Rix:** Writing – review & editing, Investigation. **Tina Leonidou:** Writing – review & editing, Investigation. **Katrin Kirsche:** Writing – review & editing, Investigation, Data curation. **Edmund Koch:** Writing – review & editing, Resources. **Achim Temme:** Writing – review & editing, Resources. **Ilker Y. Eyüpoglu:** Writing – review & editing, Resources. **Ortrud Uckermann:** Writing – review & editing, Supervision, Methodology, Conceptualization.

## Funding

Jan Rix was supported by the Faculty of Medicine Carl Gustav Carus of the TU Dresden, Germany (MeDDrive funding).

## Declaration of competing interest

The authors declare that they have no known competing financial interests or personal relationships that could have appeared to influence the work reported in this paper.

## Data Availability

Data will be made available on request.
