# Supplementary material for: Exploring the biomechanical complexity of glioblastoma spheroids and organoids with co-localized Brillouin and Raman microspectroscopy
Source: Biochem Biophys Rep. 2025 Aug 28;44:102227. doi: 10.1016/j.bbrep.2025.102227 (PMC12415078; doi:10.1016/j.bbrep.2025.102227)
Supplement: Multimedia component 1 [file mmc1.pdf]

# Exploring the biomechanical complexity of glioblastoma spheroids and organoids with co-localized Brillouin and Raman microspectroscopy

Roberta Galli [1], Jan Rix [1], Tina Leonidou [2], Katrin Kirsche [2], Edmund Koch [3], Achim Temme [2], Ilker Y. Eyüpoglu [2], Ortrud Uckermann [2]

[1] Medical Physics and Biomedical Engineering, Faculty of Medicine, TU Dresden, Dresden, Germany
[2] Department of Neurosurgery, Faculty of Medicine and University Hospital Carl Gustav Carus, TU Dresden, Dresden, Germany
[3] Clinical Sensoring and Monitoring, Department of Anesthesiology and Intensive Care Medicine, Faculty of Medicine, TU Dresden, Dresden, Germany

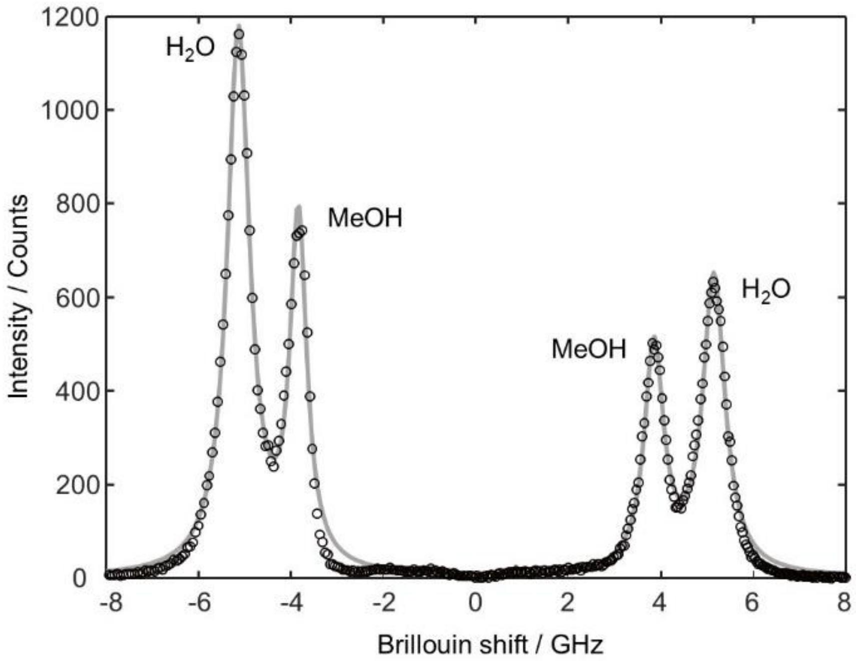


**Supp. Figure S1.** Brillouin spectrum (points) and corresponding Lorentzian fitting (gray line) acquired on water. The average distance between the spectral points confirms the calculated spectral resolution on the CCD of about 0.045 GHz.

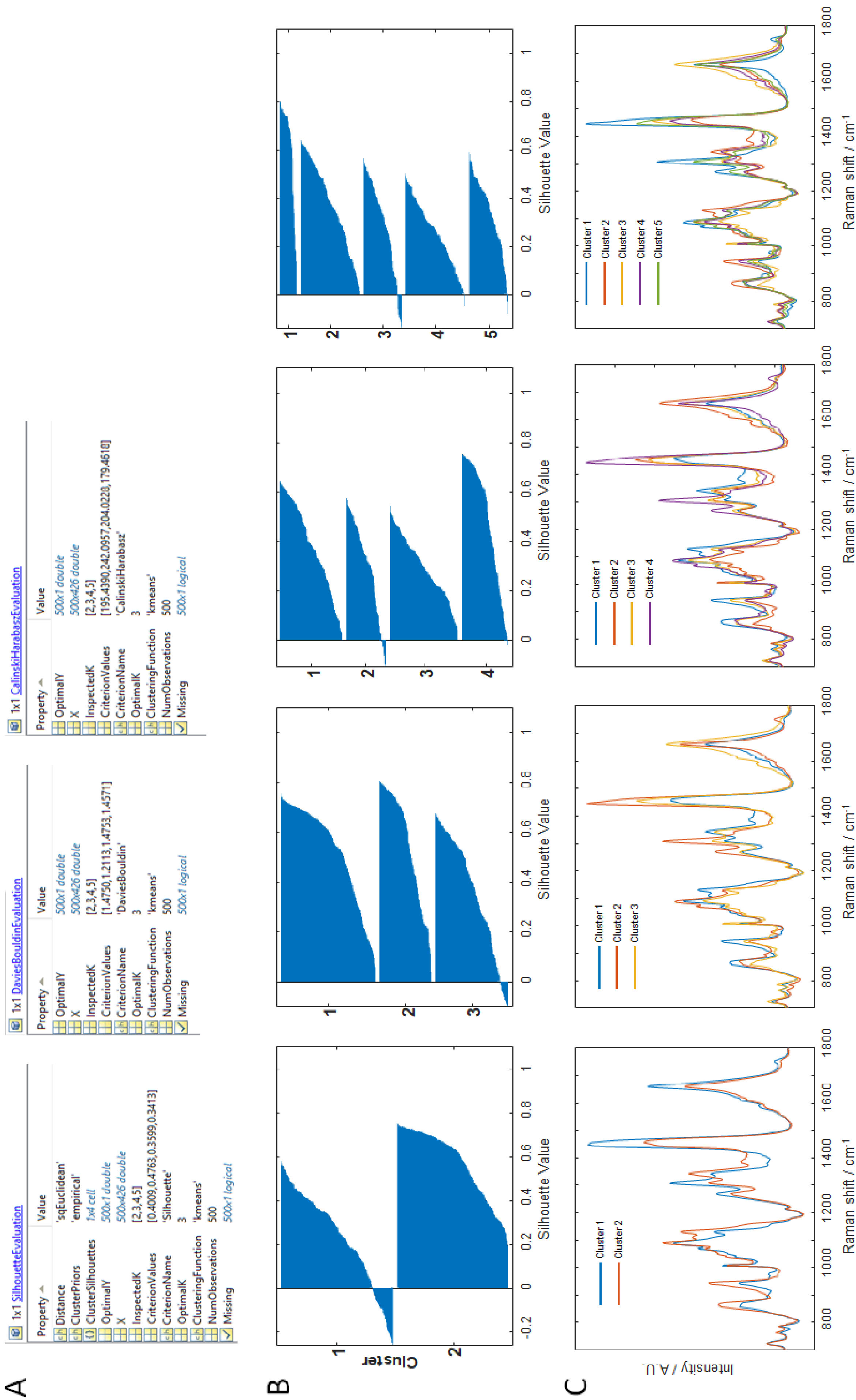
A
B
C
1x1 SilhouetteEvaluation
1x1 DaviesBouldinEvaluation
1x1 CalinskiHarabaszEvaluation
Property
Value
Cluster
Silhouette Value
Intensity / A.U.
Raman shift / cm-1
Cluster 1
Cluster 2
Cluster 3
Cluster 4
Cluster 5

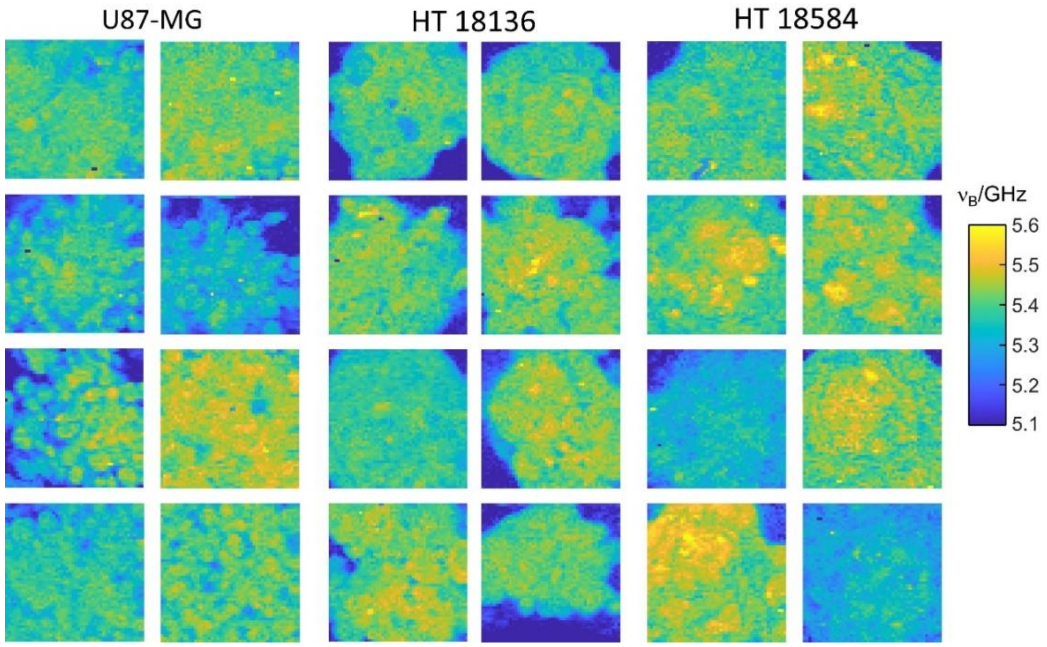


**Supp. Figure S3.** Maps of Brillouin shift of GBM spheroids; map dimension: (100 x 100) μm$^2$.

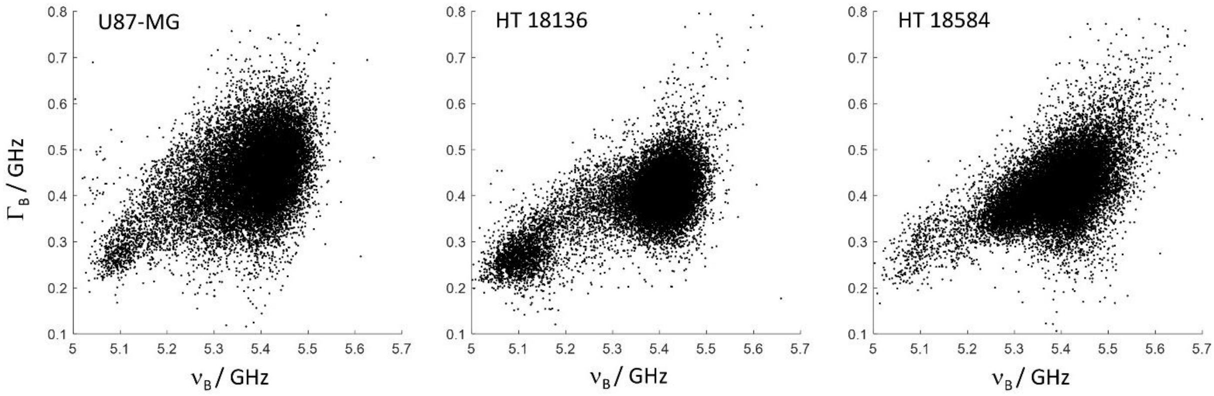


**Supp. Figure S4.** Scatter plots of Brillouin linewidth vs. shift of spheroids (for each cell line: data from 8 maps with 2500 points each).

---

**Supp. Figure S2 (at page 2). Validation of cluster number in k-means analysis of Raman spectra.** The results are shown exemplarily for the U87-MG spheroids. A: results of Matlab function "evalclusters" run with three different criteria (Silhouette, Davis-Bouldin and Calinski-Harabasz) on all acquired Raman spectra of this in vitro model; all three models gave the same answer of optimal clustering with three clusters. B: Silhouette analysis for two to five clusters, confirming the best performance with three clusters (Silhouette values > 0.6 for all three clusters, few negative values only for cluster 3). C: centroid cluster spectra for 2 to 5 clusters; clustering with only two clusters mixes the biochemical information providing spectra that contain both lipid and glycogen features overlapped to protein bands. More than three clusters provide multiple spectra that can be attributed to a basically similar biochemistry.

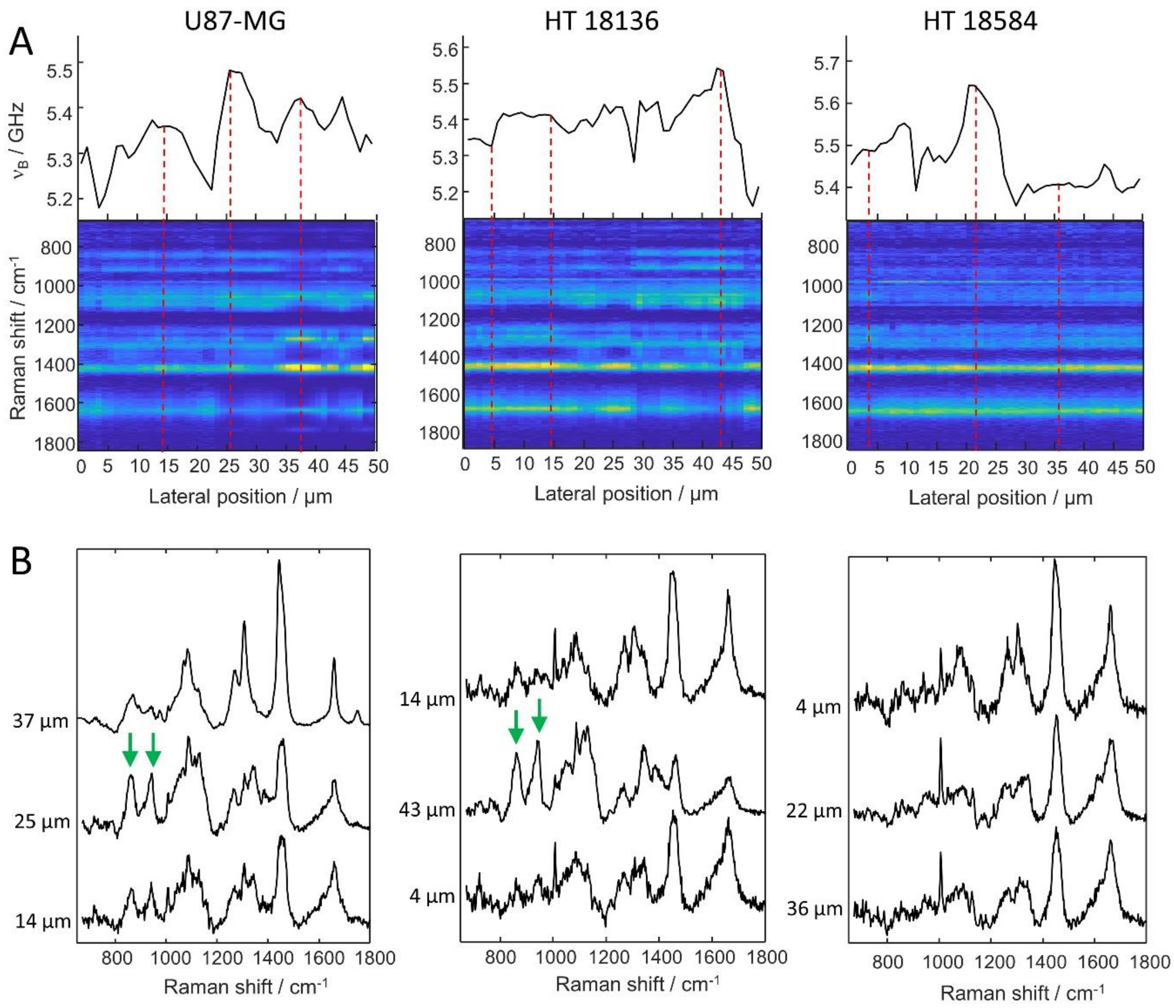


**Supp. Figure S5.** Combined Brillouin/Raman line mapping of glioblastoma spheroids. A: Brillouin shift and heat map of normalized Raman intensity along line maps; the dotted red lines indicate positions of the Raman spectra shown in B. B: Raman spectra at the selected lateral positions. For U87-MG and HT 18136 spheroids, local biochemical changes can be inferred especially from the spectral regions between 800 and 1000 $cm^{-1}$ as well as at 1440 $cm^{-1}$. Regions with very high Raman intensity at 1440 $cm^{-1}$ contain a high amount of lipids (as lateral positions 37 µm and 14 µm in the U87-MG and HT 18136 spheroids, respectively) and show an increased Brillouin shift. These regions can be related to the lipid droplets visualized by CARS in the multiphoton microscopy images of Fig. 1. The spectra at all other positions indicate proteins (as at 14 µm in the U87-MG and 4 µm in the HT 18136 spheroids) or a mixture of proteins and glycogen (as at 25 µm in the U87-MG and 43 µm in the HT 18136 spheroids). Glycogen can be specifically identified by the two Raman bands at 865 and 942 $cm^{-1}$ (green arrows), whose presence can be seen in more than half of the measurement points (see heat maps). An increased Brillouin shift was observed in the glycogen-rich regions. The HT 18584 spheroids do not contain glycogen, and the Raman spectra account for proteins (as in the positions at 22 and 36 µm) or lipids (as at 4 µm).

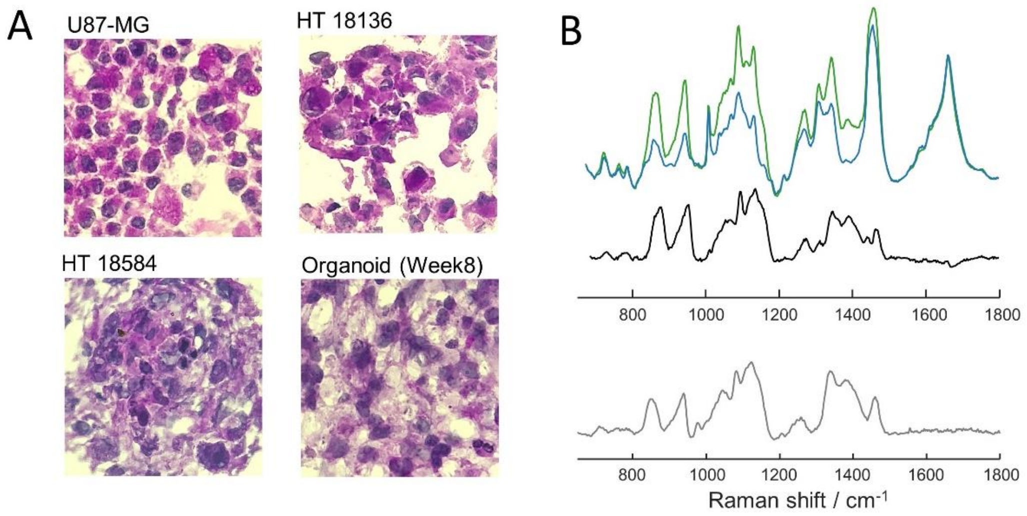


**Supp. Figure S6.** Identification of glycogen. A: PAS staining (positive: U87-MG and HT 18136; negative HT 18584 and organoid). B: Centroid Raman spectra of clusters 1 (blue) and cluster 3 (green) of U87-MG spheroids normalized to Amide I band intensity at 1650 $cm^{-1}$, difference spectra (black) obtained subtracting the spectrum of cluster 1 from the spectrum of cluster 3, and reference Raman spectrum (gray) of pure glycogen (lyophilized glycogen prod. Nr. HP51.1, Carl Roth GmbH, Germany).

**Supp. Table S1:** Comparison tests of clusters of spheroids (Dunnett's T3 multiple comparisons test for U87-MG and HT18136, Welch T-test for HT18584).

| SPHEROIDS | comparison test | $\nu_B$ P value | $\Gamma_B$ P value |
|---|---|---|---|
| **U87-MG** 1: n=166, 2: n=117, 3: n=217 | 3 vs. 2 | 0.3994 | <0.0001 |
| | 3 vs. 1 | <0.0001 | <0.0001 |
| | 2 vs. 1 | <0.0001 | <0.0001 |
| **HT18136** 1: n=326, 2: n=84, 3: n=90 | 3 vs. 2 | 0.8043 | <0.0001 |
| | 3 vs. 1 | 0.0017 | <0.0001 |
| | 2 vs. 1 | 0.0003 | <0.0001 |
| **HT18584** 1: n=403, 2: n=97 | 2 vs. 1 | 0.0531 | <0.0001 |

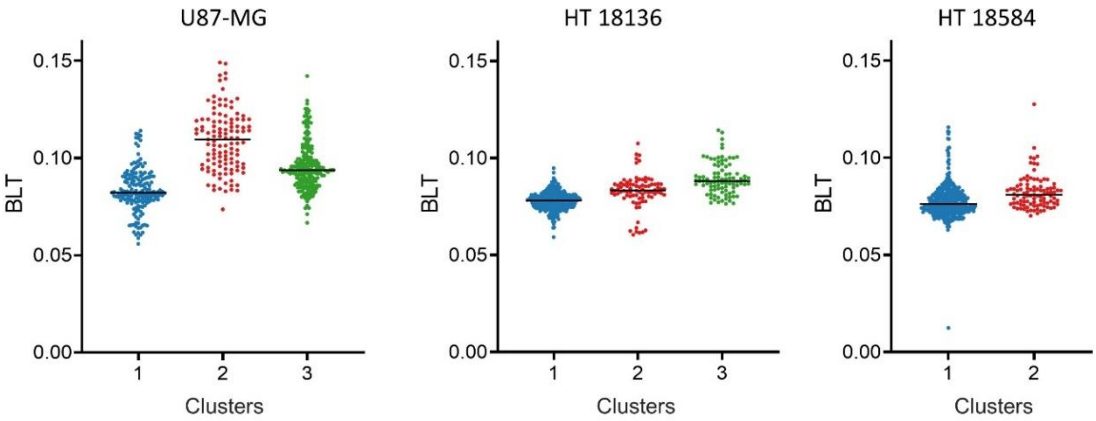


**Supp. Figure S7.** Cluster analysis of glioblastoma spheroids: Brillouin Loss Tangent (BLT).

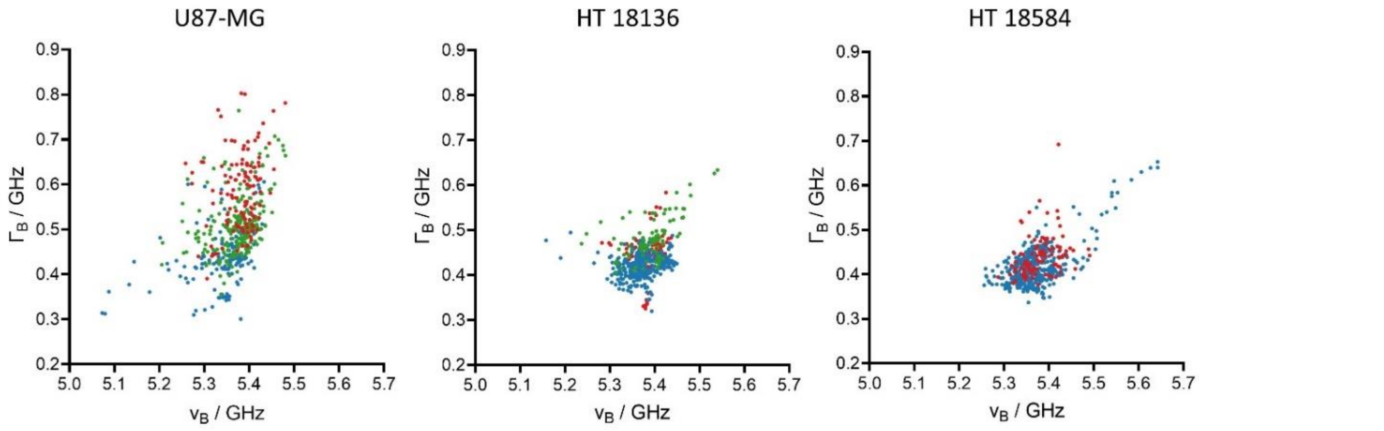


**Supp. Figure S8:** Cluster analysis of glioblastoma spheroids: Scatter plots of Brillouin linewidth vs. shift. Color coding of clusters as in Fig. 3.

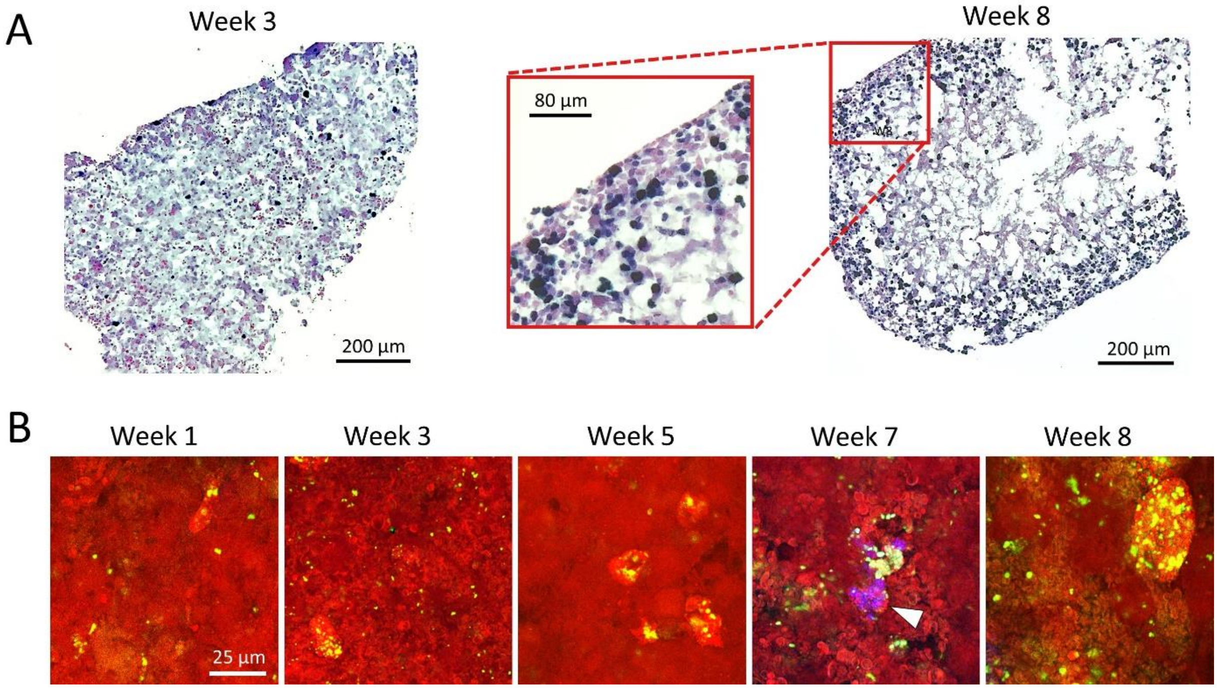


**Supp. Figure S9.** Glioblastoma organoids. A: Ki 67 histochemical staining of proliferating cells (blue) and red counterstaining of nuclei of two organoids measured at week 3 and 8. **B:** MPM images of organoids measured at selected time points. Dimension of all MPM images: 100 x 100 µm$^2$; color coding of channels: CARS: red, TPEF: green, SHG: blue. The image of the sample at week 7 shows the presence of cholesterol crystals with colocalized CARS and SHG signals (arrowhead).

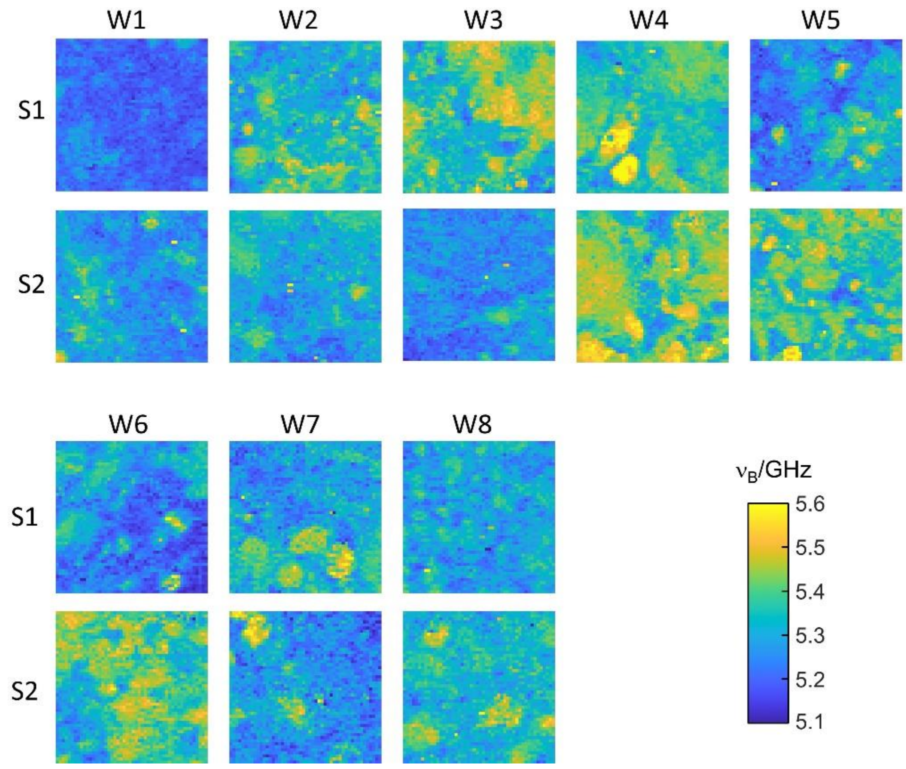


**Supp. Figure S10.** Examples of Brillouin shift maps of organoids. Two organoids (S1, S2) were measured at each time-point during eight weeks (W1…W8). Dimension of maps: 100 x 100 μm$^2$.

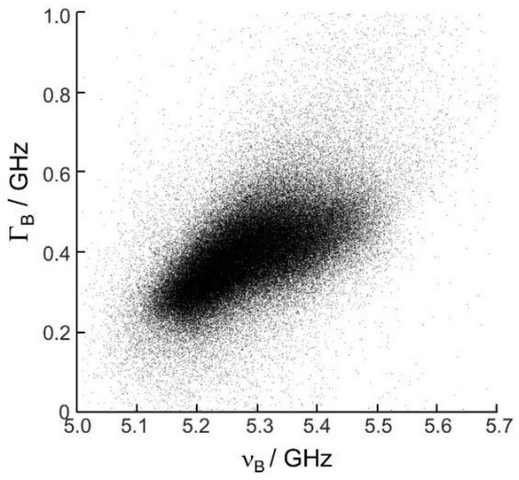


**Supp. Figure S11.** Scatter plot of Brillouin bandwidth vs. shift of glioblastoma organoids (data from 52 maps with 2500 points each).

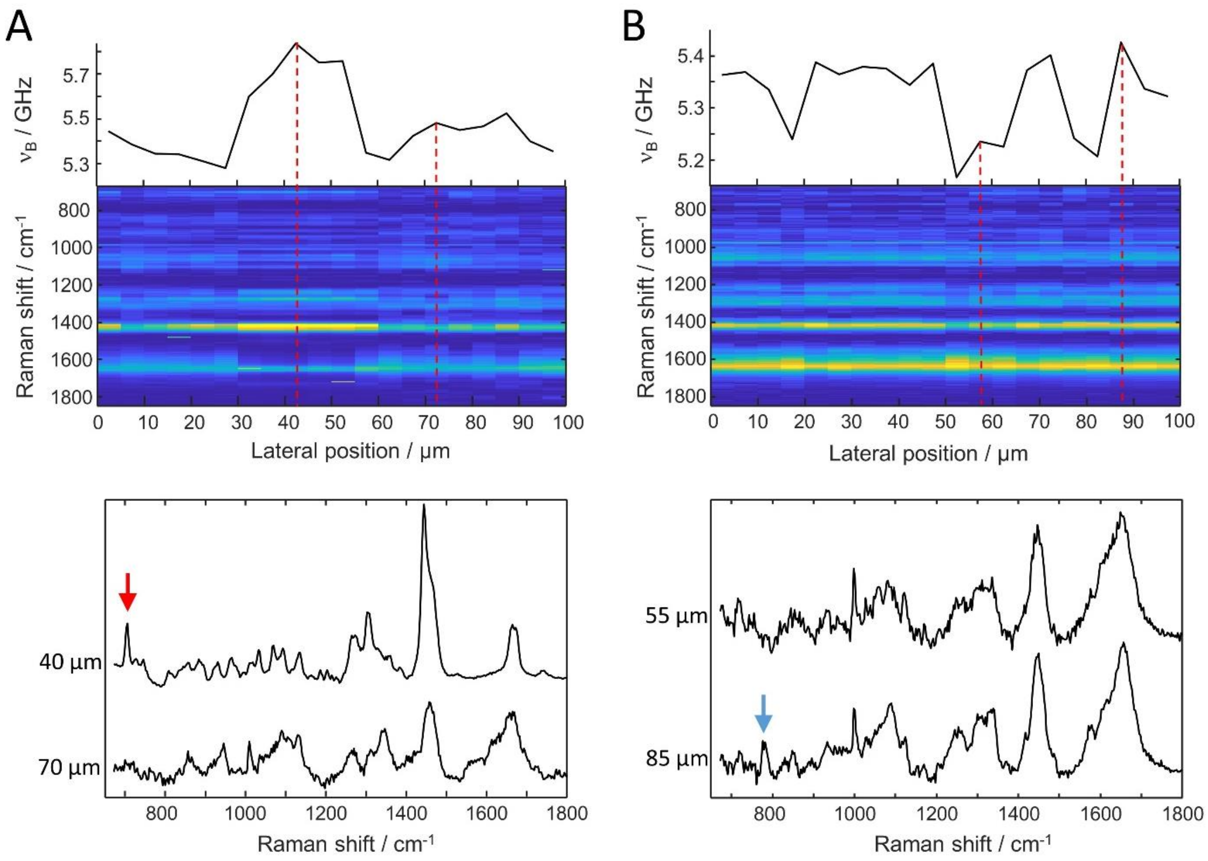


**Supp. Figure S12.** Combined Brillouin/Raman line mapping of glioblastoma organoids. Brillouin shift and heat map of normalized Raman intensity along line maps; the dotted red lines indicate positions of the Raman spectra shown below. A: Line map with local accumulation of cholesterol-rich lipids (red arrowhead: cholesterol ring vibration). They were identified with the SHG-active foam cells shown in Supporting Fig. S7 B, and correspond to strongly increased Brillouin shift ($\nu_B \approx$ 5.7 GHz) compared to tissue proteins (compare proteins at position 70 µm and lipids at 40 µm). B: Line map where cell nuclei could be differentiated from cytoplasm by analyzing the band of nucleic acids at 785 cm$^{-1}$ (compare spectrum of cytoplasmic proteins at position 55 µm and spectrum of the cell nucleus at position 85 µm, the blue arrow indicates the nucleic acid band at 785 cm$^{-1}$); the Brillouin shifts $\nu_B \approx$ 5.4 GHz for nuclei and $\nu_B \approx$ 5.25 – 5.30 GHz for cytoplasm are both in good agreement with previous experiments on single GBM cells (22).

**Supp. Table S2:** Dunnett's T3 multiple comparisons test of organoid clusters.

| **ORGANOIDS** | comparison test | $\nu_B$ P value | $\Gamma_B$ P value |
|---|---|---|---|
| Cluster 1: n = 102 | 2 vs. 3 | <0.0001 | <0.0001 |
| | 2 vs. 5 | <0.0001 | <0.0001 |
| Cluster 2: n = 59 | 2 vs. 1 | 0.0045 | 0.9860 |
| | 2 vs. 4 | 0.1176 | 0.0190 |
| Cluster 3: n = 56 | 3 vs. 5 | <0.0001 | 0.0005 |
| Cluster 4: n = 13 | 3 vs. 1 | <0.0001 | 0.0001 |
| | 3 vs. 4 | 0.0098 | 0.0070 |
| Cluster 5: n = 43 | 5 vs. 1 | <0.0001 | <0.0001 |
| Total: n = 273 * | 5 vs. 4 | 0.0027 | 0.0030 |
| | 1 vs. 4 | 0.3847 | 0.0164 |

* n = 47 measurement points were not included in the analysis, as they were attributed to a sixth cluster with centroid Raman spectrum devoid of bands (either medium or lack of Raman signal).

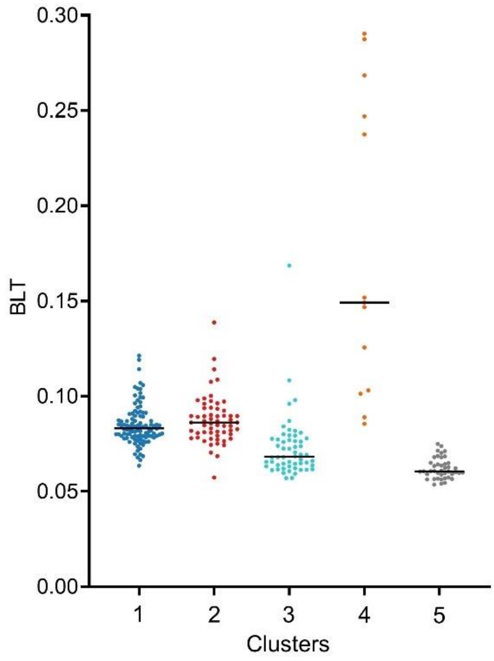


**Supp. Figure S13:** Cluster analysis of glioblastoma organoids. A: Brillouin Loss Tangent (Welch Anova test: $P < 0.001$; Dunnet's T3 multiple comparisons test: differences between groups significant except group 2 vs. 1, as already found for $\Gamma_B$ (see Supp. Table S2). B: Mean Raman spectra of the two biomechanical subgroups of cluster 4 (cholesterol-rich lipids), spectral regions showing differences are highlighted.

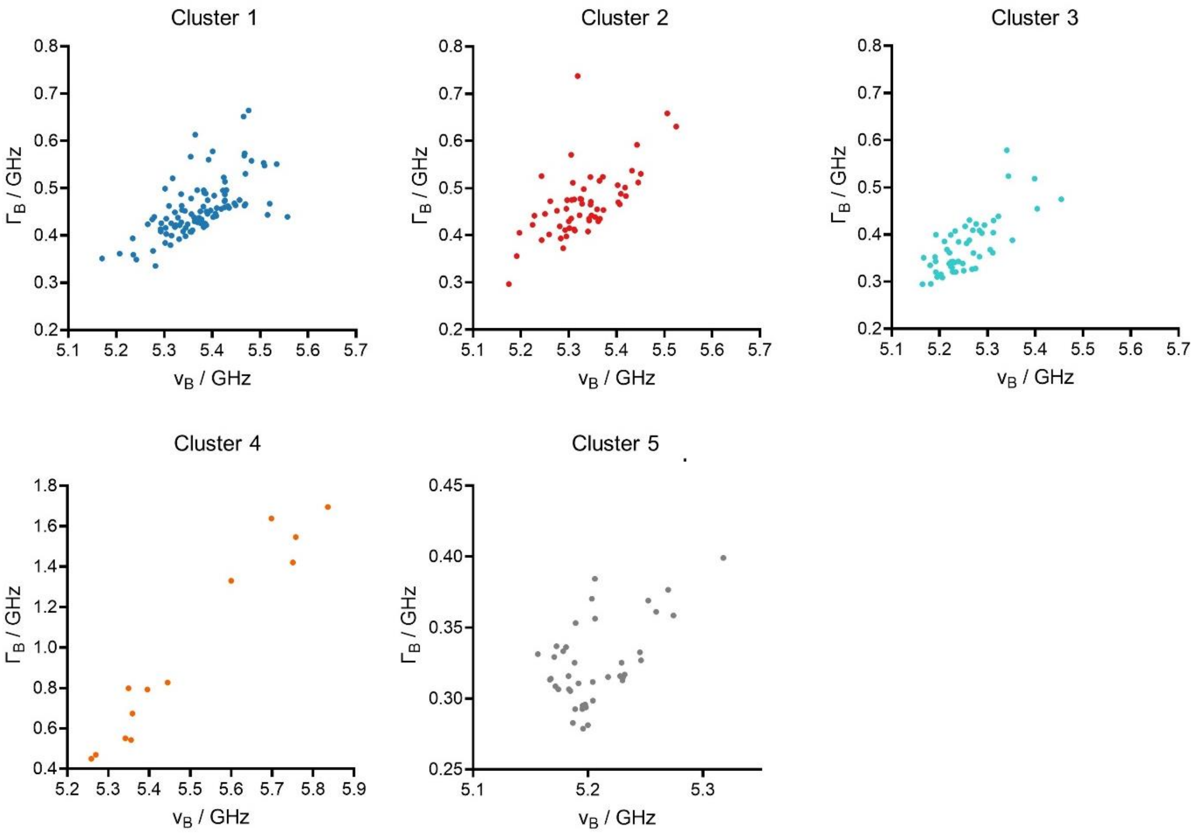


**Supp. Figure S14:** Cluster analysis of glioblastoma organoids. Scatter plot of Brillouin bandwidth vs. shift.

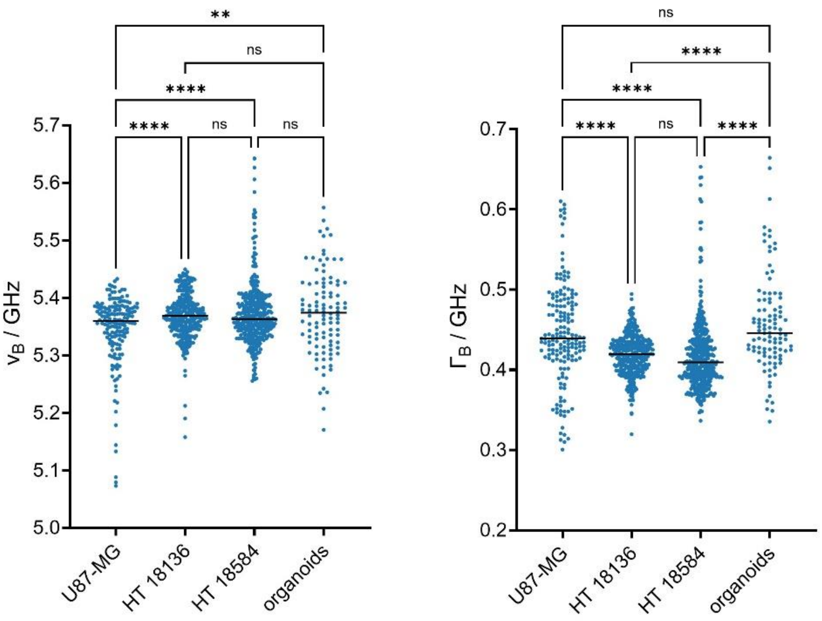


**Supp. Figure S15:** Comparison of cluster 1 (proteins) between the different models (Dunnet's T3 multiple comparisons test, ** P < 0.01, **** P < 0.0001)).
